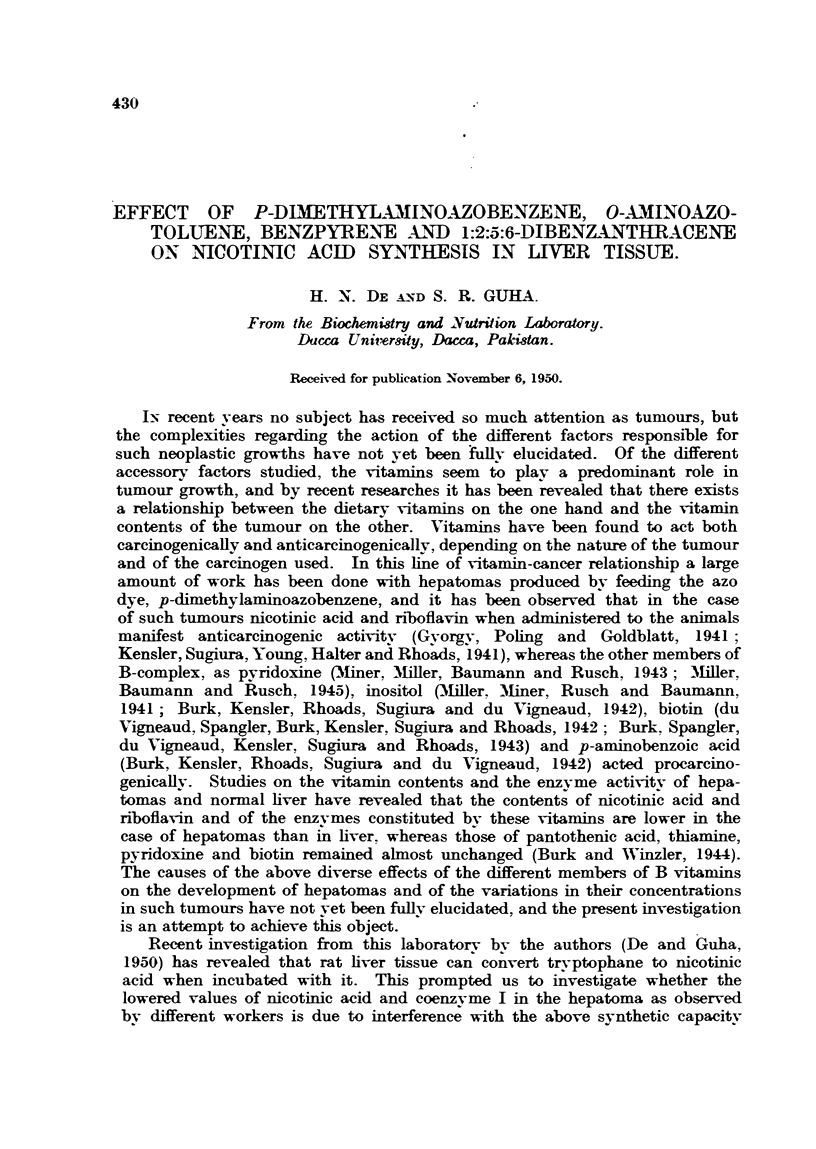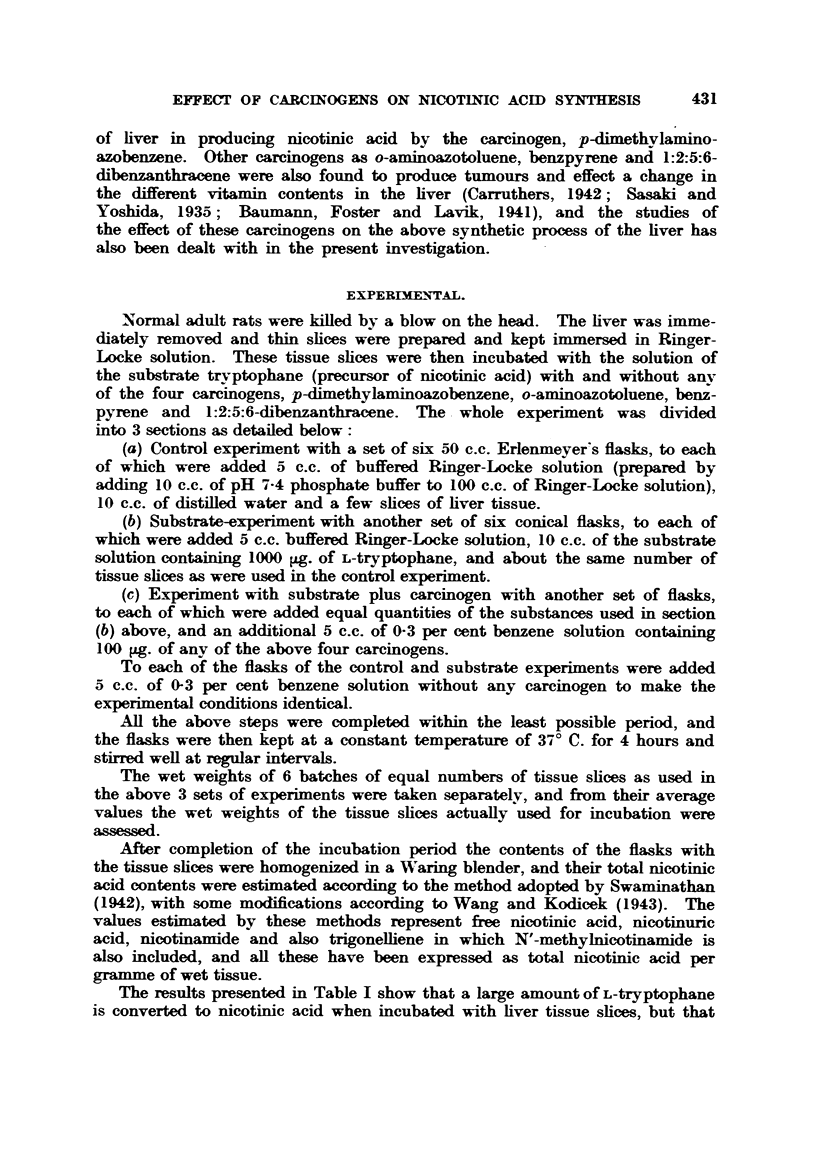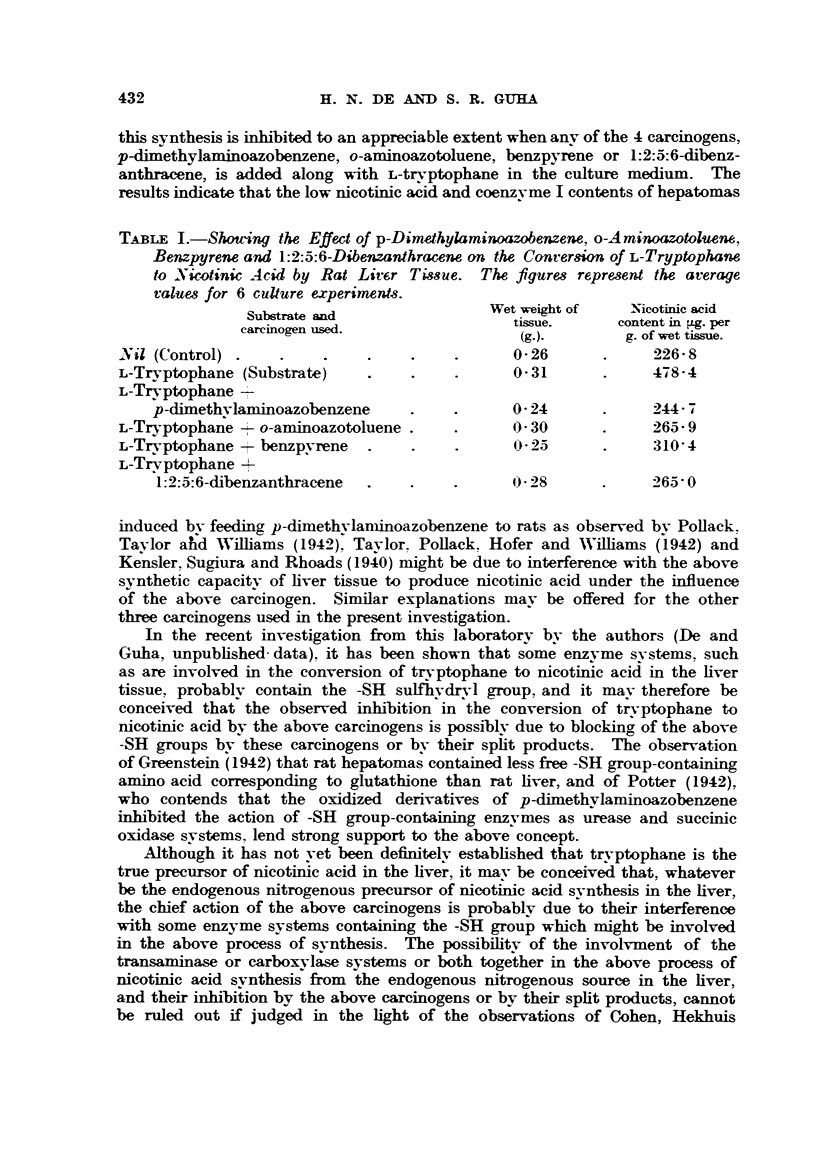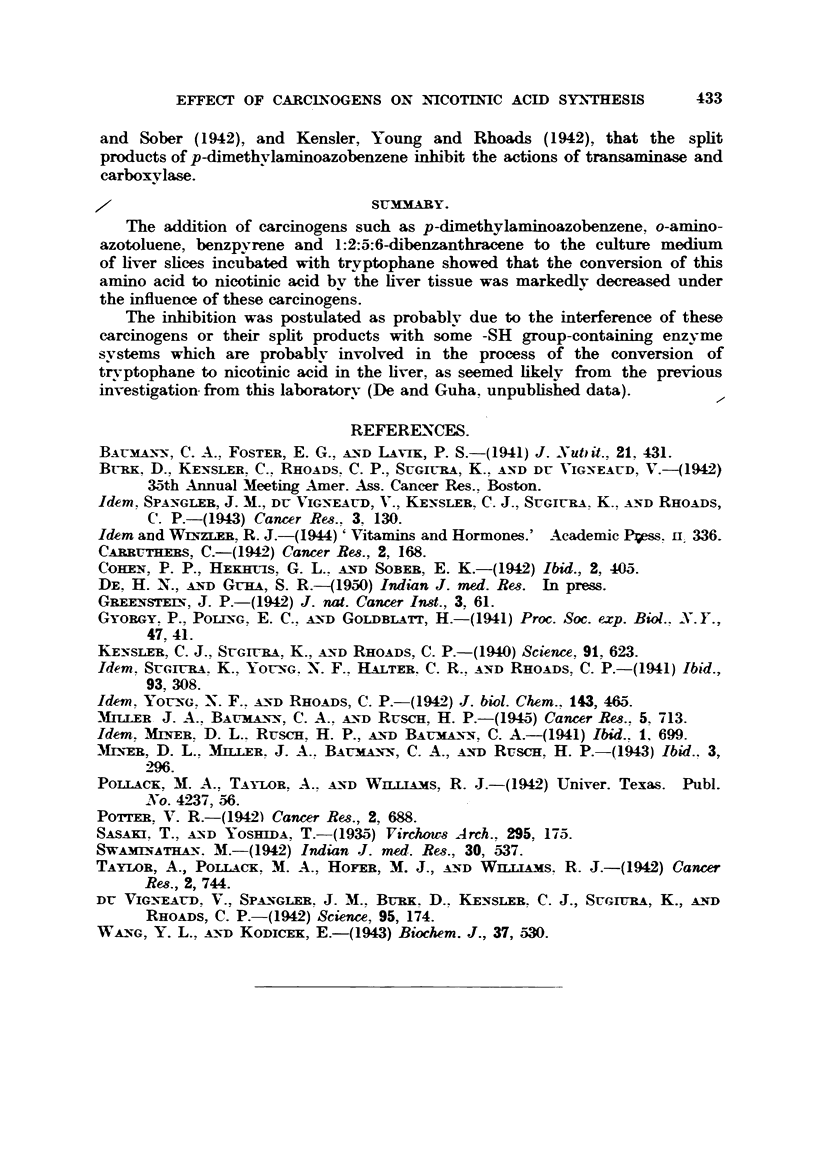# Effect of P-Dimethylaminoazobenzene, O-Aminoazotoluene, Benzpyrene and 1:2:5:6-Dibenzanthracene on Nicotinic Acid Synthesis in Liver Tissue

**DOI:** 10.1038/bjc.1950.42

**Published:** 1950-12

**Authors:** H. N. De, S. R. Guha


### EFFECT OF *P*-DIMETHYLAMINOAZOBENZENE, *O*-AMINOAZOTOLUENE, BENZPYRENE AND 1:2:5:6-DIBENZANTHRACENE ON NICOTINIC ACID SYNTHESIS IN LIVER TISSUE.

H. N. DE AND S. R. GUHA.

*From the Biochemistry and Nutrition Laboratory. Dacca University, Dacca, Pakistan.*

Received for publication November 6, 1950.

IN recent years no subject has received so much attention as tumours, but the complexities regarding the action of the different factors responsible for such neoplastic growths have not yet been fully elucidated. Of the different accessory factors studied, the vitamins seem to play a predominant role in tumour growth, and by recent researches it has been revealed that there exists a relationship between the dietary vitamins on the one hand and the vitamin contents of the tumour on the other. Vitamins have been found to act both carcinogenically and anticarcinogenically, depending on the nature of the tumour and of the carcinogen used. In this line of vitamin-cancer relationship a large amount of work has been done with hepatomas produced by feeding the azo dye, *p*-dimethylaminoazobenzene, and it has been observed that in the case of such tumours nicotinic acid and riboflavin when administered to the animals manifest anticarcinogenic activity (Gyorgy, Poling and Goldblatt, 1941 ; Kensler, Sugiura, Young, Halter and Rhoads, 1941), whereas the other members of B-complex, as pyridoxine (Miner, Miller, Baumann and Rusch, 1943 ; Miller, Baumann and Rusch, 1945), inositol (Miller, Miner, Rusch and Baumann, 1941 ; Burk, Kensler, Rhoads, Sugiura and du Vigneaud, 1942), biotin (du Vigneaud, Spangler, Burk, Kensler, Sugiura and Rhoads, 1942 ; Burk, Spangler, du Vigneaud, Kensler, Sugiura and Rhoads, 1943) and *p*-aminobenzoic acid (Burk, Kensler, Rhoads, Sugiura and du Vigneaud, 1942) acted procarcinogenically. Studies on the vitamin contents and the enzyme activity of hepatomas and normal liver have revealed that the contents of nicotinic acid and riboflavin and of the enzymes constituted by these vitamins are lower in the case of hepatomas than in liver, whereas those of pantothenic acid, thiamine, pyridoxine and biotin remained almost unchanged (Burk and Winzler, 1944). The causes of the above diverse effects of the different members of B vitamins on the development of hepatomas and of the variations in their concentrations in such tumours have not yet been fully elucidated, and the present investigation is an attempt to achieve this object.

Recent investigation from this laboratory by the authors (De and Guha, 1950) has revealed that rat liver tissue can convert tryptophane to nicotinic acid when incubated with it. This prompted us to investigate whether the lowered values of nicotinic acid and coenzyme I in the hepatoma as observed by different workers is due to interference with the above synthetic capacity of liver in producing nicotinic acid by the carcinogen, *p*-dimethylamino-azobenzene. Other carcinogens as *o*-aminoazotoluene, benzpyrene and 1:2:5:6-dibenzanthracene were also found to produce tumours and effect a change in the different vitamin contents in the liver (Carruthers, 1942; Sasaki and Yoshida, 1935; Baumann, Foster and Lavik, 1941), and the studies of the effect of these carcinogens on the above synthetic process of the liver has also been dealt with in the present investigation.

### EXPERIMENTAL.

Normal adult rats were killed by a blow on the head. The liver was immediately removed and thin slices were prepared and kept immersed in Ringer-Locke solution. These tissue slices were then incubated with the solution of the substrate tryptophane (precursor of nicotinic acid) with and without any of the four carcinogens, *p*-dimethylaminoazobenzene, *o*-aminoazotoluene, benzpyrene and 1:2:5:6-dibenzanthracene. The whole experiment was divided into 3 sections as detailed below:

(*a*) Control experiment with a set of six 50 c.c. Erlenmeyer's flasks, to each of which were added 5 c.c. of buffered Ringer-Locke solution (prepared by adding 10 c.c. of pH 7·4 phosphate buffer to 100 c.c. of Ringer-Locke solution), 10 c.c. of distilled water and a few slices of liver tissue.

(*b*) Substrate-experiment with another set of six conical flasks, to each of which were added 5 c.c. buffered Ringer-Locke solution, 10 c.c. of the substrate solution containing 1000 μg. of L-tryptophane, and about the same number of tissue slices as were used in the control experiment.

(*c*) Experiment with substrate plus carcinogen with another set of flasks, to each of which were added equal quantities of the substances used in section (*b*) above, and an additional 5 c.c. of 0·3 per cent benzene solution containing 100 μg. of any of the above four carcinogens.

To each of the flasks of the control and substrate experiments were added 5 c.c. of 0·3 per cent benzene solution without any carcinogen to make the experimental conditions identical.

All the above steps were completed within the least possible period, and the flasks were then kept at a constant temperature of 37° C. for 4 hours and stirred well at regular intervals.

The wet weights of 6 batches of equal numbers of tissue slices as used in the above 3 sets of experiments were taken separately, and from their average values the wet weights of the tissue slices actually used for incubation were assessed.

After completion of the incubation period the contents of the flasks with the tissue slices were homogenized in a Waring blender, and their total nicotinic acid contents were estimated according to the method adopted by Swaminathan (1942), with some modifications according to Wang and Kodicek (1943). The values estimated by these methods represent free nicotinic acid, nicotinuric acid, nicotinamide and also trigonelliene in which N′-methylnicotinamide is also included, and all these have been expressed as total nicotinic acid per gramme of wet tissue.

The results presented in Table I show that a large amount of L-tryptophane is converted to nicotinic acid when incubated with liver tissue slices, but that this synthesis is inhibited to an appreciable extent when any of the 4 carcinogens, *p*-dimethylaminoazobenzene, *o*-aminoazotoluene, benzpyrene or 1:2:5:6-dibenzanthracene, is added along with L-tryptophane in the culture medium. The results indicate that the low nicotinic acid and coenzyme I contents of hepatomas

TABLE I.—*Showing the Effect of* p-*Dimethylaminoazobenzene*, o-*Aminoazotoluene, Benzpyrene and* 1:2:5:6-*Dibenzanthracene on the Conversion of* L-*Tryptophane to Nicotinic Acid by Rat Liver Tissue. The figures represent the average values for* 6 *culture experiments.*

| Substrate and carcinogen used. | Wet weight of tissue. (g.). | Nicotinic acid content in µg. per g. of wet tissue. |
|---|---|---|
| *Nil* (Control) | 0·26 | 226·8 |
| L-Tryptophane (Substrate) | 0·31 | 478·4 |
| L-Tryptophane + *p*-dimethylaminoazobenzene | 0·24 | 244·7 |
| L-Tryptophane + *o*-aminoazotoluene | 0·30 | 265·9 |
| L-Tryptophane + benzpyrene | 0·25 | 310·4 |
| L-Tryptophane + 1:2:5:6-dibenzanthracene | 0·28 | 265·0 |

induced by feeding *p*-dimethylaminoazobenzene to rats as observed by Pollack, Taylor and Williams (1942), Taylor, Pollack, Hofer and Williams (1942) and Kensler, Sugiura and Rhoads (1940) might be due to interference with the above synthetic capacity of liver tissue to produce nicotinic acid under the influence of the above carcinogen. Similar explanations may be offered for the other three carcinogens used in the present investigation.

In the recent investigation from this laboratory by the authors (De and Guha, unpublished data), it has been shown that some enzyme systems, such as are involved in the conversion of tryptophane to nicotinic acid in the liver tissue, probably contain the -SH sulfhydryl group, and it may therefore be conceived that the observed inhibition in the conversion of tryptophane to nicotinic acid by the above carcinogens is possibly due to blocking of the above -SH groups by these carcinogens or by their split products. The observation of Greenstein (1942) that rat hepatomas contained less free -SH group-containing amino acid corresponding to glutathione than rat liver, and of Potter (1942), who contends that the oxidized derivatives of *p*-dimethylaminoazobenzene inhibited the action of -SH group-containing enzymes as urease and succinic oxidase systems, lend strong support to the above concept.

Although it has not yet been definitely established that tryptophane is the true precursor of nicotinic acid in the liver, it may be conceived that, whatever be the endogenous nitrogenous precursor of nicotinic acid synthesis in the liver, the chief action of the above carcinogens is probably due to their interference with some enzyme systems containing the -SH group which might be involved in the above process of synthesis. The possibility of the involvment of the transaminase or carboxylase systems or both together in the above process of nicotinic acid synthesis from the endogenous nitrogenous source in the liver, and their inhibition by the above carcinogens or by their split products, cannot be ruled out if judged in the light of the observations of Cohen, Hekhuis and Sober (1942), and Kensler, Young and Rhoads (1942), that the split products of *p*-dimethylaminoazobenzene inhibit the actions of transaminase and carboxylase.

### SUMMARY.

The addition of carcinogens such as *p*-dimethylaminoazobenzene, *o*-aminoazotoluene, benzpyrene and 1:2:5:6-dibenzanthracene to the culture medium of liver slices incubated with tryptophane showed that the conversion of this amino acid to nicotinic acid by the liver tissue was markedly decreased under the influence of these carcinogens.

The inhibition was postulated as probably due to the interference of these carcinogens or their split products with some -SH group-containing enzyme systems which are probably involved in the process of the conversion of tryptophane to nicotinic acid in the liver, as seemed likely from the previous investigation from this laboratory (De and Guha, unpublished data).

### REFERENCES.

Baumann, C. A., Foster, E. G., and Lavik, P. S.—(1941) *J. Nutrit.*, **21**, 431.

Burk, D., Kensler, C., Rhoads, C. P., Sugiura, K., and du Vigneaud, V.—(1942) 35th Annual Meeting Amer. Ass. Cancer Res., Boston.

*Idem*, Spangler, J. M., du Vigneaud, V., Kensler, C. J., Sugiura, K., and Rhoads, C. P.—(1943) *Cancer Res.*, **3**, 130.

*Idem* and Winzler, R. J.—(1944) 'Vitamins and Hormones.' Academic Press, II, 336.

Carruthers, C.—(1942) *Cancer Res.*, **2**, 168.

Cohen, P. P., Hekhuis, G. L., and Sober, E. K.—(1942) *Ibid.*, **2**, 405.

De, H. N., and Guha, S. R.—(1950) *Indian J. med. Res.* In press.

Greenstein, J. P.—(1942) *J. nat. Cancer Inst.*, **3**, 61.

Gyorgy, P., Poling, E. C., and Goldblatt, H.—(1941) *Proc. Soc. exp. Biol., N.Y.*, **47**, 41.

Kensler, C. J., Sugiura, K., and Rhoads, C. P.—(1940) *Science*, **91**, 623.

*Idem*, Sugiura, K., Young, N. F., Halter, C. R., and Rhoads, C. P.—(1941) *Ibid.*, **93**, 308.

*Idem*, Young, N. F., and Rhoads, C. P.—(1942) *J. biol. Chem.*, **143**, 465.

Miller J. A., Baumann, C. A., and Rusch, H. P.—(1945) *Cancer Res.*, **5**, 713.

*Idem*, Miner, D. L., Rusch, H. P., and Baumann, C. A.—(1941) *Ibid.*, **1**, 699.

Miner, D. L., Miller, J. A., Baumann, C. A., and Rusch, H. P.—(1943) *Ibid.*, **3**, 296.

Pollack, M. A., Taylor, A., and Williams, R. J.—(1942) Univer. Texas. Publ. *No.* 4237, 56.

Potter, V. R.—(1942) *Cancer Res.*, **2**, 688.

Sasaki, T., and Yoshida, T.—(1935) *Virchows Arch.*, **295**, 175.

Swaminathan, M.—(1942) *Indian J. med. Res.*, **30**, 537.

Taylor, A., Pollack, M. A., Hofer, M. J., and Williams, R. J.—(1942) *Cancer Res.*, **2**, 744.

du Vigneaud, V., Spangler, J. M., Burk, D., Kensler, C. J., Sugiura, K., and Rhoads, C. P.—(1942) *Science*, **95**, 174.

Wang, Y. L., and Kodicek, E.—(1943) *Biochem. J.*, **37**, 530.